# Transcriptomic and co-expression network analyses on diverse wheat landraces identifies candidate master regulators of the response to early drought

**DOI:** 10.3389/fpls.2023.1212559

**Published:** 2023-06-23

**Authors:** Liam J. Barratt, Isaac J. Reynolds, Sara Franco Ortega, Andrea L. Harper

**Affiliations:** Centre for Novel Agricultural Products (CNAP), Department of Biology, University of York, York, United Kingdom

**Keywords:** drought, transcriptomics, network analysis, triticum aestivum (bread wheat), landrace, hub gene

## Abstract

**Introduction:**

Over four billion people around the world rely on bread wheat (*Triticum aestivum* L.) as a major constituent of their diet. The changing climate, however, threatens the food security of these people, with periods of intense drought stress already causing widespread wheat yield losses. Much of the research into the wheat drought response has centred on the response to drought events later in development, during anthesis or grain filling. But as the timing of periods of drought stress become increasingly unpredictable, a more complete understanding of the response to drought during early development is also needed.

**Methods:**

Here, we utilized the YoGI landrace panel to identify 10,199 genes which were differentially expressed under early drought stress, before weighted gene co-expression network analysis (WGCNA) was used to construct a co-expression network and identify hub genes in modules particularly associated with the early drought response.

**Results:**

Of these hub genes, two stood out as novel candidate master regulators of the early drought response – one as an activator (*TaDHN4-D1*; *TraesCS5D02G379200*) and the other as a repressor (uncharacterised gene; *TraesCS3D02G361500*).

**Discussion:**

As well as appearing to coordinate the transcriptional early drought response, we propose that these hub genes may be able to regulate the physiological early drought response due to potential control over the expression of members of gene families well-known for their involvement in the drought response in many plant species, namely dehydrins and aquaporins, as well as other genes seemingly involved in key processes such as, stomatal opening, stomatal closing, stomatal morphogenesis and stress hormone signalling.

## Introduction

*Triticum aestivum* L. (bread wheat) is relied upon by billions of people as a primary source of both calories and protein (Pfeifer et al., 2014; Food and Agriculture Organization of the United Nations et al., 2018). As the global population continues to grow, the number of livelihoods that will be dependent on the success of wheat crop yields is staggering. To meet this demand, therefore, the yields of key crops like wheat need to increase by at least 50% in the coming decades (Godfray et al., 2010; Tilman et al., 2011; Ray et al., 2013). The changing climate poses a major threat to this necessary yield increase, however, with rising global temperatures leading to the depletion of water supplies and periods of intense drought stress (Hansen et al., 2006). Drier growth conditions paired with reduced water supply is of particular concern for the agricultural sector, as it accounts for between 80 and 90% of all freshwater usage, with cereal crop cultivation alone accounting for 27% (Hoekstra and Mekonnen, 2012; Ray et al., 2013; Dunn et al., 2019). In the coming decades, climate change will cause changes in precipitation patterns that may affect wheat-growing regions especially severely, with recent work finding that up to 60% of the current global wheat-growing area may face severe water scarcity by the end of the century, compared to only 15% currently (Trnka et al., 2019). As well as the threat that future drought events pose to wheat crops, drought stress has been causing significant damage around the world for the last few decades, with 161Mha of wheat harvested areas experiencing yield loss through drought between 1983 and 2009, equating to an economic loss of $47 billion (Iizumi et al., 2018; Kim et al., 2019). Therefore, the cultivation of drought tolerant wheat varieties is of paramount importance, if global wheat crops are to be protected against the effects of water shortage in a climate where water supplies are becoming increasingly scarce.

In the present work we examined the effect of drought stress exposure during early development on gene expression in spring habit wheat landrace accessions. With spring wheat often being sown during March in the Northern hemisphere, the present work mimics drought stress events that occur during April once plants have germinated and established in fields. Although much of the work concerning the effect of drought stress on wheat growth studies the perturbation’s effect on yield (Aprile et al., 2009; Zhang et al., 2018; Kim et al., 2019; Qaseem et al., 2019; Senapati et al., 2019; Abou-Elwafa and Shehzad, 2021; Lan et al., 2022; Wan et al., 2022), periods of water shortage are becoming increasingly common during the early stages of spring wheat growth, all around the world. April 2022, for example, was an incredibly dry month for many of the world’s largest wheat-producing countries, with almost 50% of the United States experiencing moderate to exceptional drought (NOAA National Centers for Environmental Information, 2022b), whilst large parts of Europe (including major spring wheat-producing nations such as the United Kingdom) experienced a drier month than normal (NOAA National Centers for Environmental Information, 2022a). The pressing nature of this threat to wheat crops is perhaps reflected in the increasing amount of research into the effect of drought stress on the early growth of wheat, over the last few years (Guo et al., 2017; Sallam et al., 2018; Ahmed et al., 2020; Ahmed et al., 2022; Mahpara et al., 2022; Nardino et al., 2022; Sharma et al., 2022). These works largely identify tolerant cultivars for use in breeding programs, but do not aim to understand the genetic control of the drought response at this stage of development – something that is relatively understudied, despite its importance (Ajigboye et al., 2017; Mao et al., 2020; Vuković et al., 2022). The need, therefore, to better understand the genetic control of the early drought response in order to aid the production of drought tolerant wheat varieties is already present, and likely to become more pressing as temperatures increase and precipitation patterns change over the coming decades.

Due to the sheer number of genes involved in complex processes, such as the drought response, identifying those which play the most pivotal roles can be difficult. The use of weighted gene co-expression network analysis (WGCNA), however, identifies groups of genes which are co-expressed across samples, from which we can identify candidate master-regulators of these groups of genes (Langfelder and Horvath, 2008; Langfelder and Horvath, 2012). Such master-regulators of drought-responsive genes, therefore, are likely to be those which play key roles in the drought response. The approach has been utilized successfully to identify “hub genes” in wheat encoding proteins such as transcription factors, heat shock proteins (HSPs) and regulators of stress hormone signaling (Lv et al., 2020; Du et al., 2022), which act to determine a plant’s degree of drought tolerance via their regulation of other drought-responsive genes. The present work employs a similar approach, but is distinct from these works due to its use of wheat landraces: genetically and phenotypically diverse cultivars selected by local farmers to grow successfully in a vast array of climates around the world (Zeven, 1998). We have previously exemplified the genetic diversity of the YoGI landrace panel, before utilizing it to identify candidate master-regulators, and genetic markers, of basal early thermotolerance (Barratt et al., 2023), but the present work represents a novel study into the use of gene expression data from wheat landraces under drought stress, to identify candidate master-regulators of the transcriptional early drought response.

## Materials and methods

### Selection, growth, and sampling of plants

14 spring habit accessions with a range of drought tolerance levels were used in the present work (**Supplementary Data S1**). Genomic tile plots visualising the A, B, and D genomes for each accession in the YoGI landrace panel (Barratt et al., 2023) were used to exclude accessions with significant genomic dominance or putative rearrangement, and to ensure all accessions used were hexaploid. Seeds were sown in Levington Advance Seed & Modular F2S compost mixed with Aggregate Industries Garside Sands 16/30 sand (80:20 ratio), treated with CaLypso insecticide (Bayer CropScience Ltd., 0.083ml mixed with 100ml water, applied to each liter of compost) and grown under long day (16/8h, 20°C/14°C) glasshouse conditions.

Four replicates of each accession per group were watered normally (twice-daily watering, average soil moisture content (SMC) = 36.6%), until plants in the drought group reached Zadoks’ growth scale 13 (GS13; Zadoks et al., 1974) whereby stress was applied by withholding water for a ten-day period. Normal watering then resumed for three-days to serve as a recovery period. Four replicates of each accession were grown at the same time, but not exposed to drought stress. All above-ground tissue from plants was harvested 13 days after GS13, before biomass was dried for two days at 70°C and weighed on a scale.

6cm of leaf tissue was collected from wheat seedlings upon reaching GS13 and at the end of the drought period. Tissue was collected individually for each sample, and immediately immersed in liquid nitrogen to prevent nucleic acid degradation. Tissue samples were stored at -80°C for later processing. At each sampling stage, as well as after drought recovery (13 days after GS13), SMC% was recorded using an ML3 Thetaprobe Soil Moisture Sensor with an HH2 Moisture Meter (Delta-T Devices, Cambridge, United Kingdom) to quantify the severity of the drought stress treatment. The probe was inserted into the soil to its full depth before moisture levels were recorded. Mean SMC% of conditions, at each time point, were compared via two-sample *t*-test.

### RNA isolation and sequencing

Total RNA was extracted from ~100 mg of individual leaf tissue samples using the E.Z.N.A Plant RNA Kit (Omega Bio-Tek, GA, USA) including a DNase treatment, according to the manufacturer’s protocol. RNA concentration was quantified using a Qubit 4 Fluorometer (Life Technologies, CA, USA), while RNA quality was assessed via both NanoDrop ND-1000 Spectrophotometer (Thermo-Fisher Scientific, MA, USA) and an Agilent Technology 2100 Bioanalyzer (Agilent Technologies, CA, USA). Samples with RNA Integrity Number (RIN) values greater than seven were deemed acceptable for use in subsequent analysis. Replicates were pooled into one sample per accession, per treatment, at equimolar proportions. Samples were stored at -80°C and shipped on dry ice to Novogene (Cambridge, United Kingdom) for sequencing, using the Illumina Novaseq 6000 platform (Illumina, CA, USA) with a 150bp paired-end strategy. Our experimental design included both technical and biological replication. Prior to sequencing, we pooled RNA from 4 replicate plants per accession, per condition (pre- or post-drought) to help control the effect of the environment on the transcriptome, whilst the different accessions provided biological replication for each treatment.

### Data processing, mapping, and quality control

After sequencing, quality control was carried out using FastQC (www.bioinformatics.babraham.ac.uk/projects/fastqc/). Raw reads were then filtered by trimming low quality sequences (average Phred score < 15), trimming short length reads (< 36bp), and clipping Illumina adapters using Trimmomatic v0.39 (Bolger et al., 2014).

Salmon (Patro et al., 2017) was used to map reads to the IWGSC *Triticum aestivum* v1.0 reference assembly (GCA Accession: GCA_900519105.1) and the updated IWGSC *Triticum aestivum* v1.1 gene model annotation. Reference genome and gene model annotation files used can be found on the International Wheat Genome Sequencing Consortium (IWGSC) website (https://www.wheatgenome.org). Salmon’s mapping-based mode was used to create an index from the reference genome, and then for quantification of the trimmed reads. Salmon output files were prepared for differential expression analysis using the R (version 4.1.2.; R R Core Team, 2021) package TxImport (version 1.24; Soneson et al., 2015), generating a table containing transcript abundance (TPM), counts, and length from the Salmon quantification files.

### Transcriptomic overview and differential expression analysis

Transcriptome data were initially explored using Principal Components Analysis (PCA) function of DESeq2 (version; 1.36.0; Love et al., 2014). Differential expression analysis was performed on the raw count data using the R package DESeq2. Genes with < 10 reads were filtered out before running DESeq2. An additive model was used to identify differentially expressed genes (DEGs) between pre- and post-drought samples. Expression fold changes were shrunk using the R package “Ashr” (version; 2.2-54; Stephens, 2017) to account for variability in lowly expressed genes while preserving large fold changes.

Only genes with a log2FoldChange greater/less than 1.5/-1.5 and an FDR-adjusted (Benjamini and Hochberg, 1995) *p*-value < 0.05 were considered significantly differentially expressed and carried forward for GO enrichment analysis. Differential expression contrasts were visualised via volcano plots, made using the “ggplot2” package (version 3.4.0; Wickham, 2009) in R.

### DEG gene ontology term enrichment analysis

To identify gene ontology (GO) terms significantly enriched amongst upregulated and downregulated DEGs, identified via DESeq2, GO enrichment analysis was conducted. Because GO terms were only present for the IWGSC RefSeqv1.0 genome annotation, we adopted an approach used previously (Borrill et al., 2019; Andleeb et al., 2023), whereby GO terms are transferred from the v1.0 annotation to the v1.1 annotation. This approach transfers the GO terms only from genes which were >99% identical across >90% of the sequence. The list of these genes can be found in Andleeb et al. (2023). IWGSC v1.0 GO terms were retrieved from: https://opendata.earlham.ac.uk/wheat/under_license/toronto/Ramirez-Gonzalez_etal_2018-06025-Transcriptome-Landscape/data/TablesForExploration/FunctionalAnnotation.rds. This RDS file was read in to R using the readRDS() function (in base R), prior to analysis.

GO terms associated with upregulated and downregulated DEGs were collated into two groups and submitted to the agriGO Singular Enrichment Analysis tool (Du et al., 2010; Tian et al., 2017). A Fisher’s exact test was performed for each DEG group with the GO terms of all genes obtained after count filtering by DESeq2 serving as background; 0.05 as the *p*-value threshold; Hochberg (FDR) as the multi-test adjustment method (Benjamini and Hochberg, 1995), and 5 as the minimum number of mapping entries threshold. A GO term was considered enriched when its FDR-adjusted *p*-value was < 0.05. GO terms that were significantly enriched amongst upregulated and downregulated genes, compared to the background, were obtained for Biological Process (BP), Molecular Function (MF), and Cellular Component (CC) categories, elucidating gene function and localisation within these DEG groups.

### Network construction and module detection

TPM data obtained from leaf tissue samples taken before and after drought stress exposure, described here, were used to construct a single co-expression network in R (version 3.6.3), using the WGCNA package (version 1.72-1; Langfelder and Horvath, 2008; Langfelder and Horvath, 2012). 21,870 genes were removed due to too many zero values, leaving 84,888 genes, from 28 samples (14 accessions before and after drought stress) for network construction. Blockwise network construction and module detection was conducted using the blockwiseModules() function according to its default parameters, with several exceptions: network type = signed hybrid, maximum block size = 5000, soft threshold power = 16 (the first power to exceed a scale-free topology fit index of 0.9), minimum module size = 30, merge cut height = 0.25. The exportNetworkToCytoscape() function was used after module detection to create edge and node files for module visualization in Cytoscape. A threshold of 0.1 was used to filter out weak connections between genes.

### Module GO term enrichment analysis

The agriGO v2.0 Singular Enrichment Analysis tool (Du et al., 2010; Tian et al., 2017) was used to identify gene ontology (GO) terms significantly enriched in each module. To do this, GO terms of genes in each module were compared to GO terms of all genes in the co-expression network. The parameters used were the same as those described for the DEG GO term enrichment analysis above. GO terms used were also retrieved using the method described above.

### DEG enrichment analysis

10,199 of the 84,888 genes included in the network were deemed to be DEGs – equating to 12% of all genes. If DEGs were distributed across modules accordingly to module size, we would expect each module to contain this proportion of DEGs. To determine whether the observed proportion of DEGs in each module was significantly greater than this predicted proportion, we used a one-proportion Z test. Modules were deemed to be significantly enriched in DEGs if *p* < 0.05.

### Network visualization and hub identification

To identify hub genes, degree (connection) scores were calculated for each gene within a module, either using the Cytoscape (version 3.9.1.; Shannon et al., 2003) network analyser tool (Assenov et al., 2008), or by counting the number of connections to and from each gene in the WGCNA edge file, using the table() function in R. The script used to calculate degree scores in R is available on GitHub (https://github.com/andreaharper/HarperLabScripts/). Cytoscape was used to visualize modules, and for hub gene identification in the majority of cases, however particularly large modules are often difficult to load, view and analyze in Cytoscape. In these cases (modules containing ~2000 genes or more), R was used to calculate degree score in the same way as in Cytoscape (i.e. counting the number of connections to and from each gene in the WGCNA edge file). Those genes in a module with the highest degree scores (most connections) were identified as the central hubs. In some cases, however, multiple genes within a module shared the highest degree score, whilst in other modules, the highest scoring genes were not found to be differentially expressed under drought conditions. In these cases, the highest-scoring DEG was identified as the module’s hub gene, as these genes are both differentially expressed and well connected within the module, and so are more likely to regulate the transcriptional drought response, than a well-connected non-DEG. Those modules found to be significantly enriched in the “response to water” (GO:0009414) GO term (black and turquoise) were also amongst the largest in the co-expression network. These modules, therefore, likely contain genes involved in diverse processes – so, to focus on the response to water, subnetworks were created using genes annotated with the “response to water” (GO:0009414) GO term within the module as guide genes. It was thought that by only examining the connections to and from these genes, the subsequently identified hub gene would be a better candidate regulator of the drought response, than the hub gene of the entire, much larger, module. As with the other modules, the most well-connected DEG was identified as the hub gene in these subnetworks.

## Results

### Drought stress exposure

**Figure 1:**
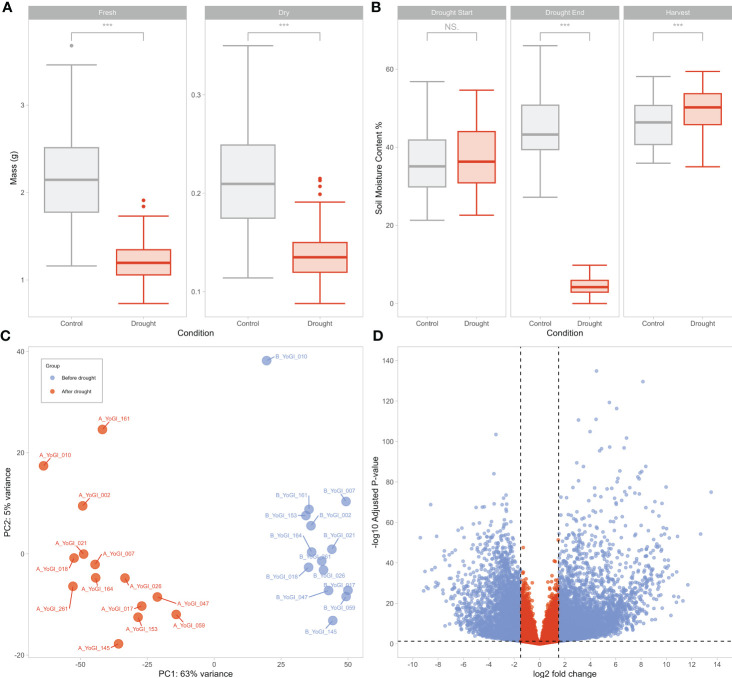
Drought treatment resulted in substantial differences across the panel in both phenotypic measurements and transcriptomic profiles. Ten days of drought stress was found to significantly reduce (*t*-test: both *p* < 2.2e-16) average fresh weight by 42.9% and average dry weight by 34.8% **(A)**, while soil moisture content (SMC%) was significantly different at the end of the drought treatment **(B)**. No significant difference was observed between control and drought groups at the start of the drought period. ‘Harvest’ refers to the end of the 3-day recovery period after the end of the drought period, where all above-ground biomass was harvested per individual. Asterisks **(A, B)** represent significance thresholds; ‘***’ represents p < 0.001. ‘N.S’ represents no significance. An initial exploration of samples and expression data suggested that samples before and after drought had distinct transcriptional profiles **(C, D)**. Principal component analysis (PCA) of variance-stabilised transcript counts from all 28 samples **(C)** showed clear separation between the two groups on PC1, while differential expression analysis identified 10,199 DEGs with differing expression before and after drought, visualised via a volcano plot **(D)**. Dashed lines indicate DEG thresholds: vertical lines represent the log2FC thresholds of ±1.5, horizontal lines represent the p-value threshold of 0.05. DEGs that meet the criteria are beyond these threshold lines, coloured in light blue. The x-axis represents the log2FC, while the y-axis represents the negative log10 of the p-value for each gene.

Drought stress was found to have a significant effect on plant growth, as both fresh and dry weight differed significantly (*t*-test: both *p* < 2.2e-16) between stressed and control plants (**Figure 1A**). Soil Moisture Content (SMC%) was measured over the course of the experiment (**Figure 1B**), with the drought stress treatment causing SMC% values of the control and drought groups to differ significantly (*p* < 2.2e-16) ten days after Zadoks’ growth scale 13 (GS13; Zadoks et al., 1974). No significant difference was identified between the two groups at GS13, before the start of the drought period (*p* = 0.179). A significant difference in SMC% was observed between the two groups at harvest (*p* = 0.0006), however. Although statistically significant, the difference in SMC% between the groups at harvest was slight, with average SMC% for both the control (45.9%) and drought (49.5%) groups being within the expected ranges for normal watering conditions. Data used to produce **Figures 1A**, **1B** are available in **Supplementary Data S2**.

### Transcriptome sequencing, quantification, and overview

921.6 Gb of raw data was generated as a result of sequencing with the Illumina paired-end Novaseq 6000 platform. From 28 samples (pooled RNA samples from 4 replicate plants, for each of the 14 accessions, before and after drought stress), 1.465 x 10^9^ reads were generated; an average of 97.3% and 92.6% of bases had a q-value of ≥ 20 and ≥ 30, respectively, with an error probability of 0.03. GC content of the reads ranged from 53.4% to 57.2%. Data quality was assessed using FastQC, with data for each sample being deemed acceptable, before pre-processing and then quantification with Salmon. Average mapping rate across all samples was 61%. Raw sequence read data were deposited in NCBI’s Gene Expression Omnibus (GSE225797).

Counts of all 28 samples were variance-stabilised using DESeq2 and analysed using principal component analysis (PCA, **Figure 1C**). The clustering of the samples indicated that the variance within each group was smaller than the variance between groups, however there was more variance on PC2 after drought than before. PC1 and PC2 accounted for 67.9% of the total variance; PC1 (which explained 62.7% of the variation) was able to provide separation between the samples taken before and after drought stress, while PC2 provided separation potentially relating to a spread of tolerance phenotypes across the accessions, albeit explaining far less of the overall variance than PC1.

### Identification and functional analysis of drought-responsive genes via differential expression and gene ontology enrichment analyses

To investigate the genes that responded to drought stress in wheat leaves during early growth stages, we carried out differential expression analysis between samples taken before and after drought stress. Genes were deemed to be differentially expressed (DEGs) when their FDR-adjusted *p*-value < 0.05, and their log2FoldChange greater/less than 1.5/-1.5.

We identified a total of 10,199 DEGs; 6051 and 4148 with significantly increased and decreased expression, respectively, in response to drought (**Figure 1D**). Wide dispersion of the genes in **Figure 1D** suggests a high level of difference in gene expression between the two groups. Normalised expression data from DESeq2 and differential expression analysis results can be found in **Supplementary Data S3**, **S4**, respectively.

To investigate gene function among DEGs, we conducted GO enrichment analysis on both the upregulated and downregulated genes. 231 GO terms were enriched amongst the upregulated genes, while 258 GO terms were enriched amongst from downregulated genes. Output from GO enrichment analyses can be found in **Supplementary Data S5**, **S6**.

GO terms related to the stress response were enriched amongst upregulated genes, such as; “response to water” (GO:0009415, FDR = 4.90e-27), “response to stress” (GO:0006950, FDR = 9.70e-11), “response to abiotic stimulus” (GO:0009628, FDR = 9.50e-16), and “response to oxidative stress” (GO:0006979, FDR = 0.0016). Other enriched terms were related to cell wall maintenance (“cell wall organization or biogenesis”, GO:1903338, FDR = 5.10e-07; “cell wall biogenesis”, GO:0042546, FDR = 2.10e-05), and regulation of gene expression and transcription (“regulation of RNA transcription, DNA-templated”, GO:0006355, FDR = 5.00e-19; “regulation of gene expression”, GO:0010468, FDR = 4.60e-18). The most significant enriched GO term was “response water”, followed by “response to acid chemical” (GO:0001101, FDR = 4.90e-27), and “oxidation-reduction process” (GO:0055114, FDR = 1.70e-23).

By contrast, GO terms enriched amongst downregulated genes were related to processes such as photosynthesis (“photosynthesis”, GO:0015979, FDR = 3.10e-76; “thylakoid”, GO:0009579, FDR = 1.20e-72; “chloroplast”, GO:0009507, FDR = 3.70e-07), homeostasis (“cellular homeostasis”, GO:0019725, FDR = 1.00e-11), and substance transport (“transport”, GO:0006810, FDR = 0.0019).

### Identifying stress-associated modules in co-expression network

The co-expression network contained 84,888 genes, housed within 81 modules (**Supplementary Data S7**). Mean module size was 1048, whilst median module size was 165. Module size ranged from 30 to 19,380 genes.

To identify modules associated with the drought response, we conducted GO enrichment analysis on each module, using all the genes included in network construction as background. We expected that modules containing genes involved in regulating the drought response would be enriched in stress-associated GO terms such as “response to water” (GO:0009414), “response to stress” (GO:0006950), or “response to abiotic stimulus” (GO:0009628). 10 of the 81 modules were significantly enriched in such GO terms (**Table 1**), with the black and turquoise modules being enriched in the GO term “response to water” (FDR = 4.8e-08 and 0.029, respectively).

**Table 1:** 10 modules were significantly enriched in GO terms related to the stress response, according to GO enrichment analysis by the AgriGO v2.0 Singular Enrichment Analysis tool (Du et al., 2010; Tian et al., 2017).

Module	Enriched GO Term
Black	Response to Water (4.8E-08)
Blue	Protein Phosphorylation (1.8E-110) Response to Stress (3.5E-06)
Cyan	Organonitrogen Compound Biosynthetic Process (5.3E-45) Response to Heat (6.2E-05)
Darkolivegreen	Protein Phosphorylation (6.3E-09) Response to Oxidative Stress (0.0023)
Magenta	Regulation of Multi-organism Process (8.7e-07) Regulation of Response to Stress (8.7e-07)
Midnightblue	Regulation of Primary Metabolic Process (0.046) Trehalose Biosynthetic Process (0.046)
Purple	Cellular Response to Stress (1.4e-06)
Salmon	Carbohydrate Metabolic Process (5.9E-10) Response to Oxidative Stress (0.031)
Tan	Phenylpropanoid Metabolic Process (1.8E-06) Response to Oxidative Stress (0.005)
Turquoise	Cellular Localization (1.5E-55) Response to Water (0.029)

The modules enriched in such GO terms are listed, as well as the most significantly-enriched GO term, and the stress-associated GO term they were also enriched in, respectively. In the instances where stress-associated GO terms were the most significantly enriched term in a module, only that term is given. The FDR-adjusted Fisher exact test p-values associated with each enriched GO term are given in brackets.

To gain further insight into which modules may contain genes particularly associated with the drought response, DEG enrichment analysis was conducted. 10,199 genes (12% of the genes included in the co-expression network) were deemed to be DEGs. If the number of DEGs was distributed across modules according to size, we would expect 12% of the genes in each module to be DEGs. We found that 17 modules contained a significantly higher proportion of DEGs than expected (**Table 2**), and so represent groups of co-expressed genes involved in the drought response – the hub genes of these modules, therefore, are promising candidates for master-regulators of the transcriptional drought response.

**Table 2:** 17 modules were significantly enriched in DEGs.

Module	Number of Genes	Observed Percentage of DEGs	*p*-value	*Mean log2-Fold Change of DEGs*
Bisque4	111	23	3.23E-04	2.99
Black	2184	21	2.22E-38	3.41
Brown	3396	69	0	-2.58
Darkolivegreen	312	49	1.91E-90	-2.62
Greenyellow	1516	20	5.37E-22	3.7
Ivory	136	71	1.91E-98	-2.76
Lightsteelblue1	163	24	1.40E-06	3.66
Mediumpurple3	165	21	0.0001	2.34
Orangered4	174	40	1.06E-30	2.87
Plum2	105	22	0.0009	2.89
Skyblue	624	27	1.54E-29	-2.85
Steelblue	512	19	3.31E-07	-2.97
Turquoise	19380	15	2.18E-32	3.1
Yellow	2709	25	6.42E-96	2.55
Darkviolet	41	22	0.025	-2.39
Grey60	1024	14	0.027	-2.72
Salmon	1433	15	9.10E-05	3.23

These modules contained a significantly higher proportion of DEGs than expected should the total number have been distributed across modules according to their size (12%). These modules are listed, as well as the number of genes in each module, the proportion of these genes which were observed to be DEGs, the *p*-value result from the one-proportion Z-test, and the mean log2-fold change values of the DEGs within each module.

Combined, these analyses identified modules which were particularly stress-associated, either as a result of the enrichment of stress-associated GO terms, or the enrichment of DEGs. Only hub genes from those modules listed in **Tables 1**, **2**, therefore, were examined further to determine whether they may be promising candidate master-regulators of the transcriptional early drought response.

### Hub gene identification

The hub genes within those modules deemed to stress-associated (**Tables 1**, **2**) may act as master-regulators of the transcriptional drought response, as they are significantly co-expressed with many stress-associated and/or drought-responsive genes. These hub genes (**Table 3**) seemingly play roles in diverse processes, such as stress hormone signalling (*TraesCS6A02G340100* and *TraesCS4D02G325200*) or the biotic stress response (*TraesCS5A02G052600* and *TraesCSU02G171500*). One hub gene, meanwhile, was found to be drought-responsive in the present work, but is likely a key actor in photosynthesis, and so is probably required to aid growth and development under normal conditions (*TraesCS6D02G247400*), whereas others were completely uncharacterized and do not share sequence identity with any well understood gene (*TraesCS3D02G361500*, *TraesCS4D02G251500, TraesCS4A02G212000, and TraesCS4A02G190700*), making their potential role as regulators of the drought response completely novel. Modules which were particularly large likely contained genes involved in diverse processes. Some of the largest modules were also significantly enriched in the “response to water” (GO:0009414) GO term, therefore to identify candidate master-regulators of processes of interest (namely, the drought response) subnetworks were created using genes annotated with this GO term as guide genes. This was done for the black and turquoise modules, with the subsequent subnetworks’ hub genes (*TraesCS5D02G379200* and *TraesCS6D02G234700*, respectively) being identified as dehydrins.

**Table 3:** Hub genes identified in stress-associated modules may be strong candidates for master-regulators of the drought response, based on their high number of connections to other genes within stress-associated modules.

Hub Gene	Module	Log2FC	BLAST Hit	Putative Function	Reference
*TraesCS4D02G251500*	Bisque4	1.99	*Aegilops tauschii subsp. strangulata B3 domain-containing protein Os03g0212300*	Uncharacterized	
*TraesCS5D02G379200*	Black	5.87	*TaDHN4-D1*	Drought tolerance and drought response	(Hao et al., 2022)
*TraesCS5D02G194500*	Blue	2.04	*Aegilops tauschii subsp. strangulata senescence-induced receptor-like serine/threonine-protein kinase*	Senescence	(Shin et al., 2019)
*TraesCS6D02G247400*	Brown	-2.26	*T. aestivum phosphoribulokinase, chloroplastic-like*	Calvin Cycle, Response to salt stress	(Xu et al., 2016; Yu et al., 2020a)
*TraesCS5A02G087200*	Cyan	-1.64	*Triticum aestivum psbP domain-containing protein 1, chloroplastic-like*	Photosystem I assembly factor	(Liu et al., 2012)
*TraesCS5A02G052600*	Darkolivegreen	-3.28	*Triticum aestivum probable glucan 1,3-beta-glucosidase A*	Response to fungal pathogen	(Münch-Garthoff et al., 1997)
*TraesCS2D02G127000*	Darkviolet	-2.02	*Triticum aestivum quinone-oxidoreductase QR2-like*	Protection against oxidative stress	(Greenshields et al., 2005)
*TraesCS4A02G212000*	Greenyellow	5.23	*Triticum aestivum uncharacterized LOC123082151*	Uncharacterized	
*TraesCS7A02G034500*	Grey60	-3.76	*TaGSTU6*	Cold tolerance	(Lv et al., 2022)
*TraesCS3D02G361500*	Ivory	-3.75	*T. aestivum uncharacterized LOC123079795*	Uncharacterized	
*TraesCSU02G171500*	Lightsteelblue1	2.97	*Triticum aestivum esterase PIR7B-like*	Biotic stress response	(Wäspi et al., 1998)
*TraesCS2A02G129200*	Magenta	1.67	*Triticum aestivum cytochrome b561 and DOMON domain-containing protein At5g47530-like*	Electron transport	(Asard et al., 2013)
*TraesCS5A02G477300*	Mediumpurple3	2.01	*Triticum aestivum zinc finger protein ZAT8-like*	Regulation of programmed cell death	(Feng et al., 2023)
*TraesCS3D02G144500*	Midnightblue	3.3	*Triticum aestivum protein RICE FLOWERING LOCUS T 1-like*	Flowering activator	(Komiya et al., 2008; Komiya et al., 2009; Ogiso-Tanaka et al., 2013)
*TraesCS6A02G340100*	Orangered4	2.23	*Triticum urartu ethylene-responsive transcription factor ERF018-like*	Regulation of ethylene and ABA signalling	(Chen et al., 2016)
*TraesCS7D02G220700*	Plum2	2.45	*Triticum aestivum probable serine/threonine-protein kinase PBL7*	Regulation of brassinosteroid signalling	(Nolan et al., 2017)
*TraesCS4A02G462000*	Purple	1.5	*Triticum aestivum noroxomaritidine synthase 2-like*	Noroxomaritidine synthesis	(Singh and Desgagné-Penix, 2017)
*TraesCS2D02G224200*	Salmon	10.36	*Triticum aestivum isocitrate lyase*	Glucnoegenesis, Salt tolerance	(Runquist and Kruger, 1999; Yuenyong et al., 2019)
*TraesCS1A02G314800*	Skyblue	-2.73	*Triticum aestivum high molecular mass early light-inducible protein HV58, chloroplastic-like*	Cold tolerance	(Lee et al., 2020)
*TraesCS4A02G190700*	Steelblue	-1.84	*Triticum aestivum uncharacterized LOC123082090*	Uncharacterized	
*TraesCS2D02G518200*	Tan	1.74	*Triticum aestivum tryptophan decarboxylase 1-like*	Serotonin biosynthesis	(Kang et al., 2009)
*TraesCS6D02G234700*	Turquoise	2.43	*Triticum aestivum dehydrin COR410-like (COR410)*	Cold tolerance	(Danyluk et al., 1994; Danyluk et al., 1998)
*TraesCS4D02G325200*	Yellow	1.65	*A. tauschii subsp. strangulata serine/threonine-protein kinase BSK1-2*	Regulation of brassinosteroid signalling	(Nolan et al., 2017)

Each hub gene’s module membership and log2FC are given, as well as their identity and putative function.

Hub genes in these stress-associated modules (**Table 3**) represent valuable targets for further inquiry into the regulation of the transcriptional drought response, and as targets for breeders in for the production of drought tolerant varieties. However, two of these hub genes, *TraesCS5D02G379200 (TaDHN4-D1)* and *TraesCS3D02G361500* (uncharacterised gene), were deemed to be particularly promising candidates as master-regulators of both the transcriptional and physiological drought responses, due to the likely functions of the genes they were connected to in the co-expression network. *TraesCS5D02G379200* may regulate the expression of a suite of fellow dehydrins, as well as stress-responsive transcription factors and genes which may affect stomatal dynamics – all of which show significant up-regulation of expression under drought stress. *TraesCS3D02G361500* may also regulate the expression of genes likely involved in controlling stomatal dynamics, as well as other potentially guard cell-localized genes involved in stomatal morphogenesis, and several aquaporins – however, unlike *TraesCS5D02G379200*, the hub, and the genes it is connected to, are downregulated significantly under drought stress.

## Discussion

### Utilizing landraces to future-proof wheat crops

It is widely believed that landraces are an important genetic resource available to breeders for the production of more climate-resilient wheat varieties, thanks to their extensive phenotypic and genetic diversity (Zeven, 1998; Reynolds et al., 2007; Corrado and Rao, 2017; Schmidt et al., 2019; Cseh et al., 2021; Tehseen et al., 2022). This diversity has been extensively exploited in grass crops such as rice and barley, with many landrace accessions either being screened for drought tolerance (Van Oosterom et al., 1993; Tardy et al., 1998; Munasinghe et al., 2017; Dbira et al., 2018; Kumar et al., 2019; Mishra et al., 2019; Boudiar et al., 2020; Sabouri et al., 2022; Bakhshi and Shahmoradi, 2023), utilized to identify the genetic determinants of drought tolerance (Yu et al., 2012; Fan et al., 2015; Reinert et al., 2016; Hoang et al., 2019; Beena et al., 2021), or used to better understand the drought response (Cantalapiedra et al., 2017; Khodaeiaminjan et al., 2023). Wheat landraces, however, remain relatively underutilized in the study of drought tolerance and the drought response (Dodig et al., 2012; Lin et al., 2019; Naderi et al., 2020; Gómez-Espejo et al., 2022). After highlighting both its extensive genetic diversity, and its usefulness in the study of early thermotolerance (Barratt et al., 2023), here we show the YoGI landrace panel can also be used to effectively study the response to early drought stress, and aid the production of drought tolerant wheat varieties.

The effect of drought stress on yield is well studied (Zhang et al., 2018; Kim et al., 2019; Qaseem et al., 2019; Senapati et al., 2019; Abou-Elwafa and Shehzad, 2021; Lan et al., 2022; Wan et al., 2022), but as the climate continues to change, periods of water shortage coinciding with the early growth stages of spring wheat crop growth are likely to become more common around the world. There has already been evidence of this, with major spring wheat-producing countries such as the USA and the UK experiencing drier than average periods in the months after spring wheat sowing (NOAA National Centers for Environmental Information, 2022a; NOAA National Centers for Environmental Information, 2022b). The majority of the work examining the effect of drought stress on wheat seedling growth has not aimed to identify regulators of the drought response during this early stage of development, however (Guo et al., 2017; Sallam et al., 2018; Ahmed et al., 2020; Ahmed et al., 2022; Mahpara et al., 2022; Nardino et al., 2022; Sharma et al., 2022) – something that remains relatively understudied (Ajigboye et al., 2017; Mao et al., 2020; Vuković et al., 2022). The present work, therefore, takes a novel approach to elucidate how the early drought response is transcriptionally controlled in wheat landraces, and represents a promising step towards the production of more drought tolerant varieties.

### Drought stress causes substantial changes in the wheat transcriptome

Our analysis demonstrates that the expression profiles of spring wheat are vastly different before and after drought; over 10,000 genes were differentially expressed between the two groups. GO term enrichment analysis of DEGs indicated that growth and development was deprioritized; DEGs annotated with photosynthesis-, and chlorophyll-related GO terms were largely downregulated. Similarly, there was widespread downregulation of genes annotated with enriched Cellular Component GO terms such as “thylakoid”, suggesting a reduction in light-dependent reactions. Photosynthetic regulation is associated with both oxidative and drought stress responses; stress-related changes in photosynthetic activity under various environmental stress conditions have been identified in other cereals such as rice (Gan et al., 2019; Yu et al., 2020b), as plants seek to limit damage to critical components. Downregulation of genes involved in photosynthesis under drought is common among grasses, with studies in *Miscanthus* (De Vega et al., 2021), *Brachypodium distachyon* (Priest et al., 2014), and rice (Liang et al., 2021) demonstrating similar trends.

Reduced photosynthetic activity can result in an excess of absorbed light energy, inducing the generation of toxic reactive oxygen species (ROS; Pospíšil, 2016). GO enrichment analyses conducted on both up- and downregulated DEGs identified a number of enriched GO terms (such as “response to oxidative stress”) involved in both the production and mitigation of ROS and other oxidative agents. Both up- and down-regulation of genes involved in cellular oxidation and reduction has previously been observed in other grasses, like rice (Sirohi et al., 2020). ROS accumulation, while promoting immune responses and stomatal guard cell closure (Song et al., 2014), can also cause oxidative damage to DNA and photosynthetic machinery, potentially leading to cell death (Huang et al., 2019; Ye et al., 2021). DEGs annotated with such terms were primarily identified as peroxidases and oxidases; their presence among both up- and downregulated DEGs is likely due to their cellular localization, mediating ROS accumulation in some tissues over others (Csiszár et al., 2012).

Our GO enrichment of the upregulated genes identified a number of DEGs annotated with drought- and osmotic-stress enriched GO terms. These genes included a variety of dehydrins and other late embryogenesis abundant (LEA) genes, known key actors in various abiotic stress responses in wheat (Kosová et al., 2014; Hassan et al., 2015; Liu et al., 2019). Studies in species such as *B. distachyon* and *O. sativa* were similarly able to identify an upregulation of dehydrins (Smita et al., 2013; Sancho et al., 2022), suggesting that this is a common response among grasses.

Downregulation of genes under the term “transport”, which included genes involved in water transport processes, likely facilitated the conservation of water for critical organelles and guard cells, as well as mediating water loss by decreasing membrane permeability (Maurel et al., 2008; Patel and Mishra, 2021).

These trends in the expression of stress and growth-associated genes indicate a shift towards stress-mitigation, often seen with abiotic stresses such as harsh drought (Zhang et al., 2020).

### *TaDHN4* may regulate the expression of dehydrins and drought tolerance genes under drought stress

**Figure 2:**
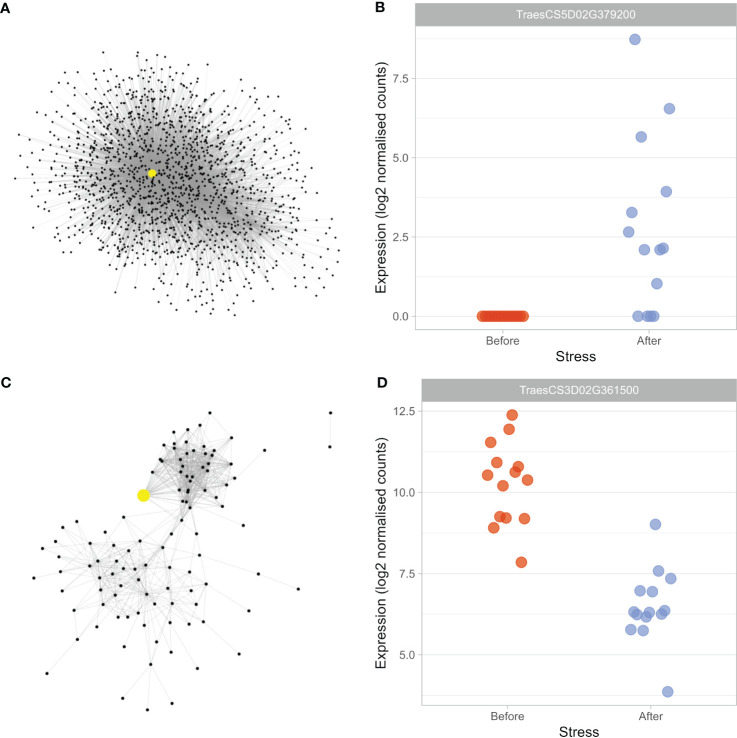
Drought-associated modules house candidate master-regulators of the early drought response. To focus on genes likely involved in the drought response within the large black module, a subnetwork was created **(A)**, whereas the ivory module **(C)** was small enough to be analysed in its entirety. The hub genes within the black subnetwork (TraesCS5D02G379200) and ivory module (TraesCS3D02G361500) are highlighted in yellow and enlarged. Expression of TraesCS5D02G379200 **(B)** was found to be significantly upregulated (log2FC = 5.87) in response to drought stress, whereas TraesCS3D02G361500 **(D)** expression was significantly downregulated (log2FC = -3.75).

The black module was significantly enriched in DEGs (**Table 2**), as well as the GO term “response to water” (FDR-adjusted *p*-value = 4.8E-08, **Table 1**), suggesting the module houses genes which play key roles in the drought response. Due to the size of the module (2184 genes), it is likely to contain genes involved in various processes besides the drought response. To focus on those genes most likely to play a role in the drought response, a subnetwork was created using the genes within the module which possessed the significantly enriched GO term “response to water” as guide genes. The subnetwork contained 1544 genes, and 6562 connections between genes (**Figure 2A**).

The central hub gene was *TraesCS5D02G379200*, which possessed the enriched GO term “response to water” and was connected to 1222 other genes in both the full module (where it had the sixth highest degree score) and the subnetwork. The gene shares 100% sequence identity with *Aegilops tauschii subsp. strangulata dehydrin DHN2*, but has been classed as *TaDHN4-D1* in recent work (Hao et al., 2022). We found that expression of the gene was upregulated significantly (log2FC = 5.87) after drought stress (**Figure 2B**); consistent with the commonly observed expression responses of dehydrins in response to drought stress (Sun et al., 2021; Tiwari and Chakrabarty, 2021). *TaDHN4* belongs to the YSK_2_ sub-family of dehydrins (Wang et al., 2014), a sub-family shown to increase stress tolerance when overexpressed in *Arabidopsis* (Brini et al., 2007) and whose expression, consistent with the present work, was most strongly upregulated in dehydrated leaves of wheat seedlings (Wang et al., 2014). Four of the five most well-connected genes in the subnetwork were homeologues, or duplicates, of *TaDHN4*; *TraesCS5D02G379200* (hub, *TaDHN4-D1), TraesCS5B02G372100* (*TaDHN4-B1*), *TraesCS5B02G372200* (*TaDHN4-B2*) and *TraesCS5A02G369900* (*TaDHN4-A2*) – suggesting both that all homeologues share similar expression responses, and that there is likely functional redundancy amongst the homeologues, meaning they may all play roles in regulating the drought response.

Further support for the hypothesis that the hub gene may act as a master-regulator of the drought response comes from the genes it is connected to in the subnetwork. We found that the hub was connected to 220 DEGs in the subnetwork, 62.3% of all DEGs within it. Amongst these DEGs were several other members of the dehydrin family, besides the hub’s homeologues and duplicates to which it was also connected: *TraesCS5B02G426800* (log2FC = 9.89) encodes *T. aestivum dehydrin Rab15-like*, whilst *TraesCS6A02G350600* (log2FC = 8.39) is *T.aestivum dehydrin DHN3-like*. The hub gene is also connected to other genes with different functions related to the drought response: *TraesCS2D02G364500* (log2FC = 8.83), and its homeologue *TraesCS2A02G367700*, are *T. aestivum chromosome D caleosin (Clo10)* – a member of another drought-responsive gene family thought to be involved in the drought response, via action on stomatal aperture and transpiration (Aubert et al., 2010; Kim et al., 2011). The hub may also have far-reaching effects on global gene expression, due to its connection to drought-responsive transcription factors such as *TaNAC29*, *TraesCS2A02G367700* (log2FC = 7.32), which has been shown to increase drought and salinity tolerance when expressed in *Arabidopsis* (Huang et al., 2015). The hub’s connection to drought-responsive genes with these kinds of functions further suggests that it may act as a master-regulator of the drought response.

Dehydrins act as molecular chaperones to maintain protein structure and functional folding under stressful cellular conditions, so the hub gene’s ability to regulate gene expression may not be immediately apparent. Recent evidence, however, suggests that there are multiple potential mechanisms by which dehydrins can control the expression of other genes. This can occur as a result of their chaperone activity, protecting transcription factors and other transcriptional regulators from damage by cellular stress, ensuring their function and subsequent effect on gene expression is maintained (Tiwari and Chakrabarty, 2021). There is also emerging evidence that dehydrins themselves may act as transcription factors, with rice lines overexpressing *OsDhn-Rab16D* showing increased expression of ABA signalling and stress-responsive genes (Tiwari et al., 2019). Dehydrins may also effect gene expression by binding directly to DNA and protecting it from damage by ROS during stress events; this is not a commonly-observed role played by dehydrins, however, only being reported in grape and citrus (Hara et al., 2009; Boddington and Graether, 2019). Each of these roles would rely on the hub gene protein being localized in the nucleus, but, according to recent work, the hub gene appears to be localized to the cytoplasm (Hao et al., 2022). In the present work, we have seen evidence that the hub gene dehydrin may act to control the expression of other drought-responsive dehydrins, as well as several other stress-responsive genes which seemingly play roles in the drought response, suggesting either the hub gene may in fact be localized to the nucleus under drought stress, or that the protection it provides transcriptional regulators in the cytoplasm is sufficient to allow them to act functionally once translocated to the nucleus.

### Uncharacterized hub gene potentially controls stomatal dynamics, water movement and stress hormone signaling under drought stress

The ivory module (**Figure 2C**) was identified as drought-associated, as it was significantly enriched in DEGs (**Table 2**). The most well-connected gene in the module was *TraesCS3D02G361500*, with its homeologues (*TraesCS3A02G368600* and *TraesCS3B02G400100*) also amongst the top five most well-connected genes in the module. Expression of the hub gene, *T. aestivum uncharacterized LOC123079795*, was found to be downregulated under drought stress (log2FC = -3.75, **Figure 2D**), suggesting the gene may play a repressive role during the transcriptional and physiological drought responses.

35 of the 41 genes the hub was connected to were also DEGs, all of which were downregulated under drought stress, with several having functions related to the drought response. *TraesCS1A02G070200* (log2FC = -4.79) is *T. aestivum jasmonate-induced oxygenase 1-like*, and also shared some sequence identity (69%) to a large region of its *Arabidopsis* namesake, and orthologue (identified using Ensembl Plants; Yates et al., 2022), *AtJOX1*. The gene is a negative regulator of jasmonic acid (JA) signaling, conducting hydroxylation of JA, inactivating it in the signaling pathway (Caarls et al., 2017). JA is known to accumulate in plant cells during drought stress and increase tolerance to drought stress in wheat (Wasternack, 2014; Ali and Baek, 2020; Wang et al., 2021). JA has also been shown to act in unison with ABA to control stomatal closure in *Arabidopsis* (Hossain et al., 2011), suggesting the hub gene may be able to determine stomatal aperture via control over *TraesCS1A02G070200* expression, and subsequently, JA signaling.

The hub gene is also connected to several other DEGs potentially involved in regulating stomatal opening. *AtAO1* plays a role in programmed cell death via its production of reactive oxygen species, as well as a role in protoxylem differentiation in root tissue (Møller and McPherson, 1998; Ghuge et al., 2015a; Ghuge et al., 2015b), and is the *Arabidopsis* orthologue (identified using Ensembl Plants; Yates et al., 2022) of *TraesCS4B02G282700* (log2FC = -4.61) which encodes *T. aestivum primary amine oxidase 1-like*. As well as this, *AtAO1* expression was found to be both induced by methyl-jasmonate, and localized in guard cells, and other tissues involved in regulating water homeostasis – leading the authors to suggest that the gene may play a key role in regulating stomatal closure (Ghuge et al., 2015b). Previous work suggests *AtAO1* promotes stomatal closure, however here we see *TraesCS4B02G282700* expression being downregulated under drought stress, suggesting it may act to repress stomatal closure in wheat. *TraesCS4A02G398700* (log2FC = -4.2) was also connected to the hub gene, and similarly may play role in stomatal dynamics. The gene is *T. aestivum GDSL esterase/lipase APG-like*, whilst also sharing sequence identity (66%) with large regions of *AtGGL19*, a gene found to be expressed in *Arabidopsis* guard, pavement and mesophyll cells, whose expression was also downregulated under drought stress, suggesting the gene may play a role in stomatal closure (Xiao et al., 2021). These observations, paired with the downregulation of *TraesCS4A02G398700* under drought stress, suggest the gene may act to repress stomatal closure. *TraesCS1B02G176000* was another downregulated DEG (log2FC = -3.96) connected to the hub gene, and encodes *T. aestivum cytokinin dehydrogenase 3-like*. The gene appears to also be involved in stomatal biology, as a result of its inactivation of cytokinins. However, overexpression of *TraesCS1B02G176000*’s *Arabidopsis* namesake, *AtCKX3*, improved drought tolerance in tomato and *Arabidopsis* thanks to reduced transpiration, likely from reduced leaf area and stomatal density (Werner et al., 2010; Farber et al., 2016). The downregulation of *TraesCS1B02G176000* in the present work, however, suggests it may act to increase water loss, unlike its *Arabidopsis* namesake. Despite the gene’s name, *TraesCS1B02G176000* showed the highest level of sequence identity to *AtCKX6* – a guard cell-localized gene with a potential role in stomatal morphogenesis (Werner et al., 2003). Because of this, and its downregulation under drought stress in the present work, we suggest that *TraesCS1B02G176000* may play a positive role in stomatal morphogenesis, as reducing the production of stomata under drought stress is likely to limit the amount of water loss via transpiration (Bertolino et al., 2019).

Two genes involved in water transport were also connected to the hub. *TraesCS4D02G024400*, *T. aestivum protein NRT1/PTR FAMILY 8.3-like*, was downregulated under drought stress (log2FC = -3.34) and shares sequence identity (63%) with a large region of its namesake, *AtNPF8.3*. The gene appears to play a role in water uptake in germinating *Arabidopsis* seeds, as knockout mutant seeds showed a 17% lower water content compared to WT (Choi et al., 2020). *TraesCS4B02G310900* (log2FC = -1.74) also appears to be involved in water transport, as it is *T. aestivum aquaporin TIP1-1-like*, but shares marginally more sequence identity with *AtTIP2* (73%) than *AtTIP1* (72%). The downregulation of these genes under drought stress in the present work, paired with their membership of a module containing so many potential guard cell-localized genes, suggests that these genes may act to control guard cell turgidity, via their control of water movement in and out of the cells. When guard cells are turgid, stomata are open, whilst flaccid guard cells cause stomata to close – suggesting that the downregulation of these water uptake genes in response to drought stress may be a mechanism to cause stomatal closure, and prevent excess moisture loss under water shortage. Recent work has shed light on the relationship between water uptake proteins, such as aquaporins, and stomatal dynamics (Grondin et al., 2015; Ding and Chaumont, 2020a; Ding and Chaumont, 2020b; Cui et al., 2021), suggesting the hub may act to reduce water loss via its downregulation of these water uptake genes under drought stress.

Here, we present the YoGI landrace panel as a valuable resource for the study of the transcriptional control of the drought response, and useful tool for breeders in the development of climate-resilient wheat varieties. We identified thousands of genes differentially expressed before and after exposure to drought stress during early development. The use of co-expression network analysis allowed us to identify several hub genes which may act as master-regulators of the transcriptional early drought response. Two very promising candidate hub genes, however, may act to coordinate both the transcriptional and physiological early drought responses, as they potentially control the drought-responsive expression of stress-associated genes such as dehydrins, aquaporins and genes involved in stomatal dynamics. Further work is required, however, to make the link between the potential action of these hub genes on drought-responsive gene expression, and the physiological drought response.

## Data availability statement

The datasets presented in this study can be found in online repositories. The original data is publicly available at NCBI, GSE225797.

## Author contributions

LB, IR and AH conceived and planned the project. LB and IR performed plant growth experiments and RNA extraction. IR and SF performed transcriptome data mapping. LB and IR conducted transcriptomic analyses. LB, IR and AH wrote the manuscript, and all authors reviewed it. All authors contributed to the article and approved the submitted version.
